# Supplementary material for: Microstructure Evolution in Plastic Deformed Bismuth Telluride for the Enhancement of Thermoelectric Properties
Source: Materials (Basel). 2022 Jun 14;15(12):4204. doi: 10.3390/ma15124204 (PMC9230931; doi:10.3390/ma15124204)
Supplement: Supplementary file 1 [file materials-15-04204-s001.zip › materials-1755184-supplementary.pdf]

Supplementary Material

Microstructure evolution in plastic deformed bismuth telluride for the enhancement of thermoelectric properties

Haishan Shen[1], In-Yea Kim[2], Jea-Hong Lim[2], Hong-Baek Cho[1], Yong-Ho Choa[1,*]

[1]Department of Materials Science and Chemical Engineering, Hanyang University, 55 Hanyangdaehak-ro, Sangnok-gu, Ansan, Gyeonggi-do 15588, Republic of Korea.

[2]Department of Materials Science and Engineering, Gachon University, 1342 Seongnamdaero, Sujeong-gu, Seongnam-si, Gyeonggi-do 13120, Republic of Korea.

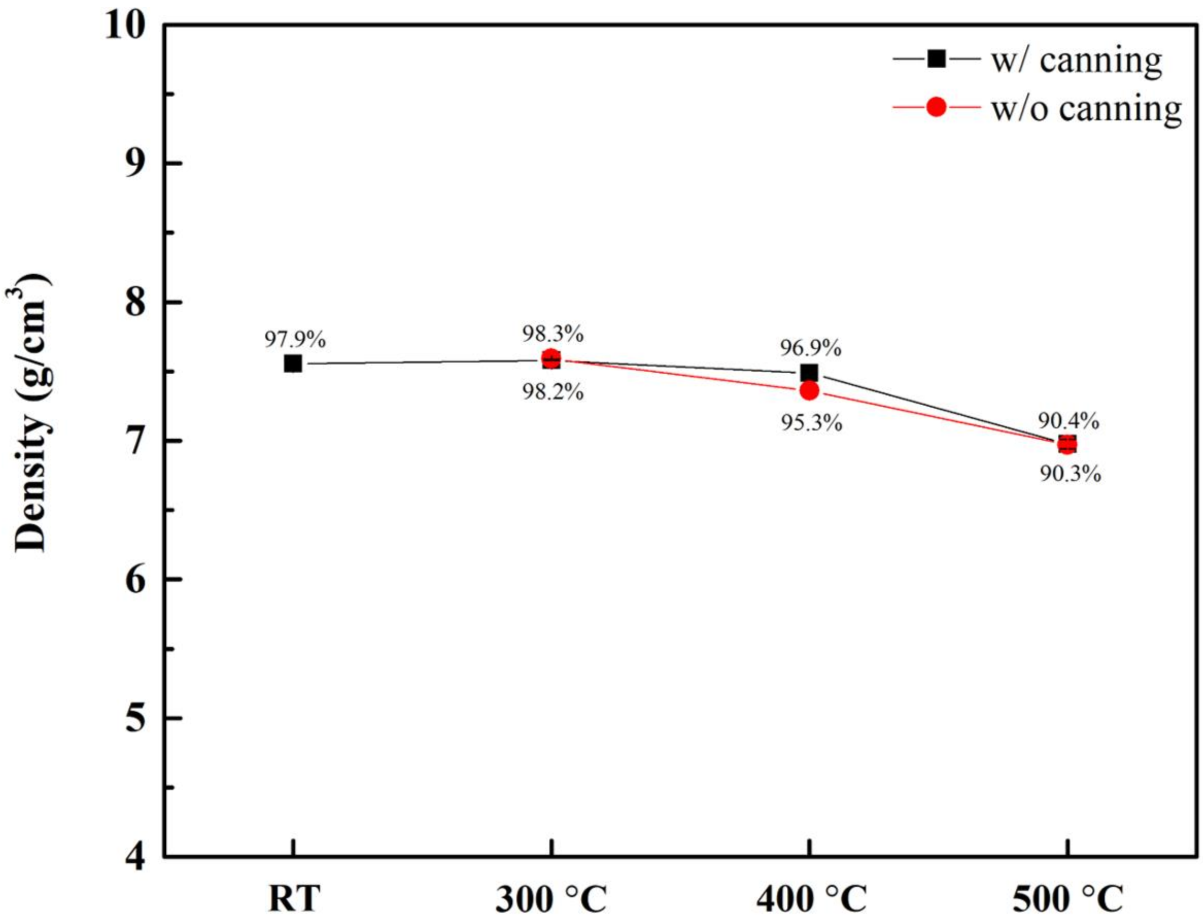

Figure S1. Comparison of pellet densities at RT, 300, 400, and 500 °C.

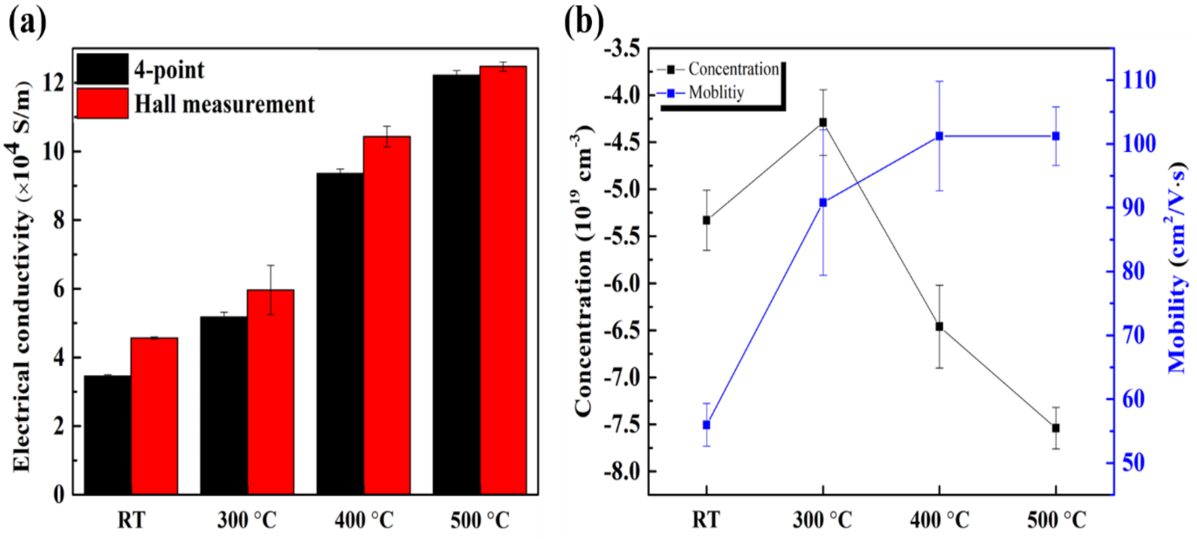

Figure S2. (a) comparison of 4-point and hall measurement about electrical conductivity and (b) carrier concentration and mobility from hall measurement.

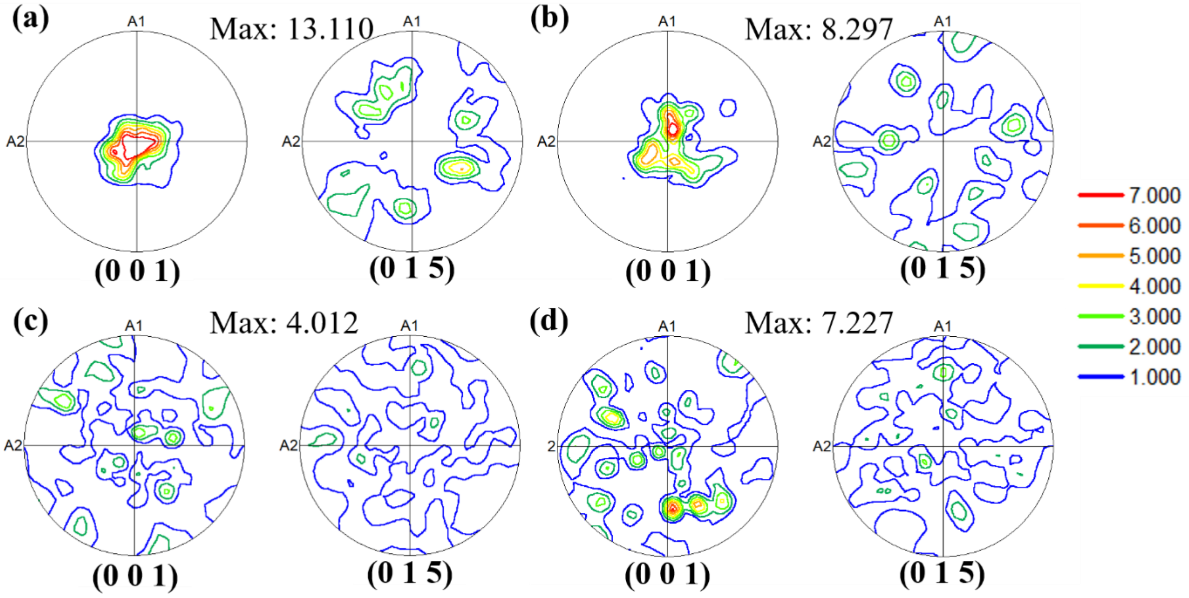

Figure S3. EBSD pole figure images of samples for (0 0 1) and (0 1 5) planes at (a) RT, (b) 300 ℃, (c) 400 ℃, and (d) 500 ℃. Electron diffraction intensity is represented by color marks in scale bar.

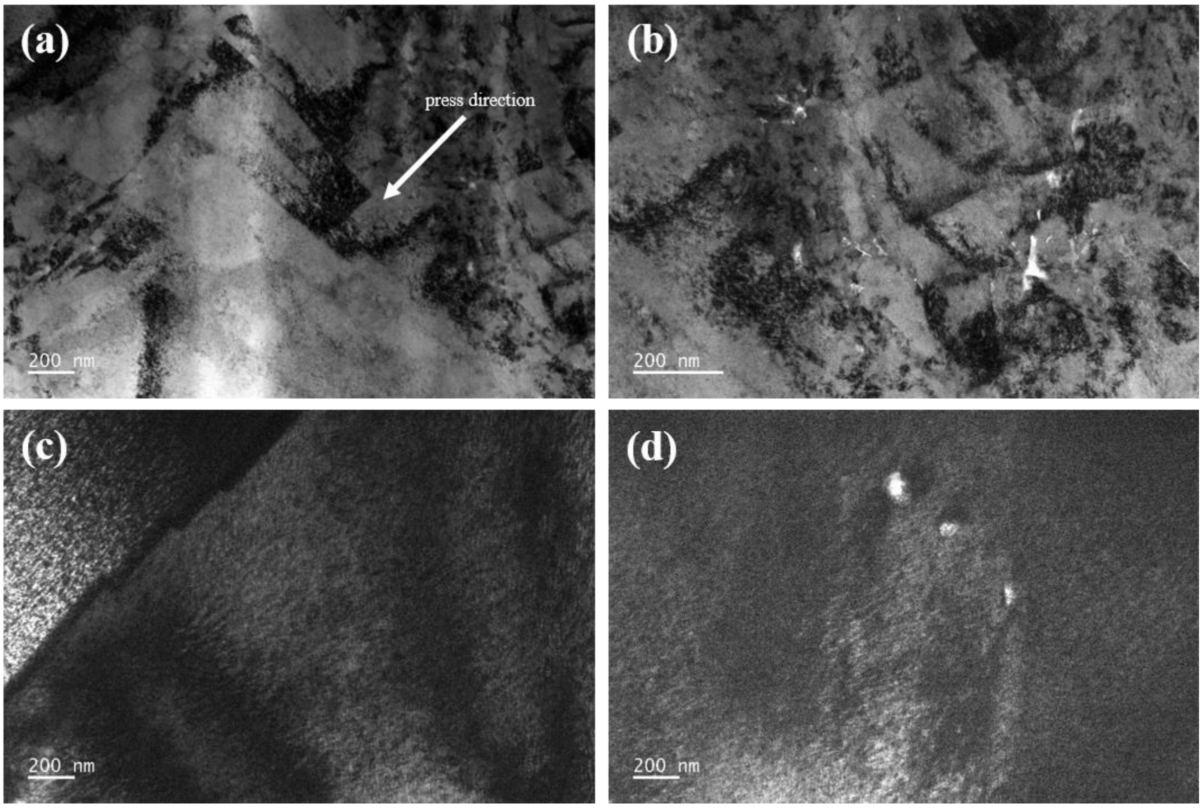

Figure S4. TEM images of cold-sintered samples (a–b) at RT and (c–d) annealed at 500 ℃ with canning package.

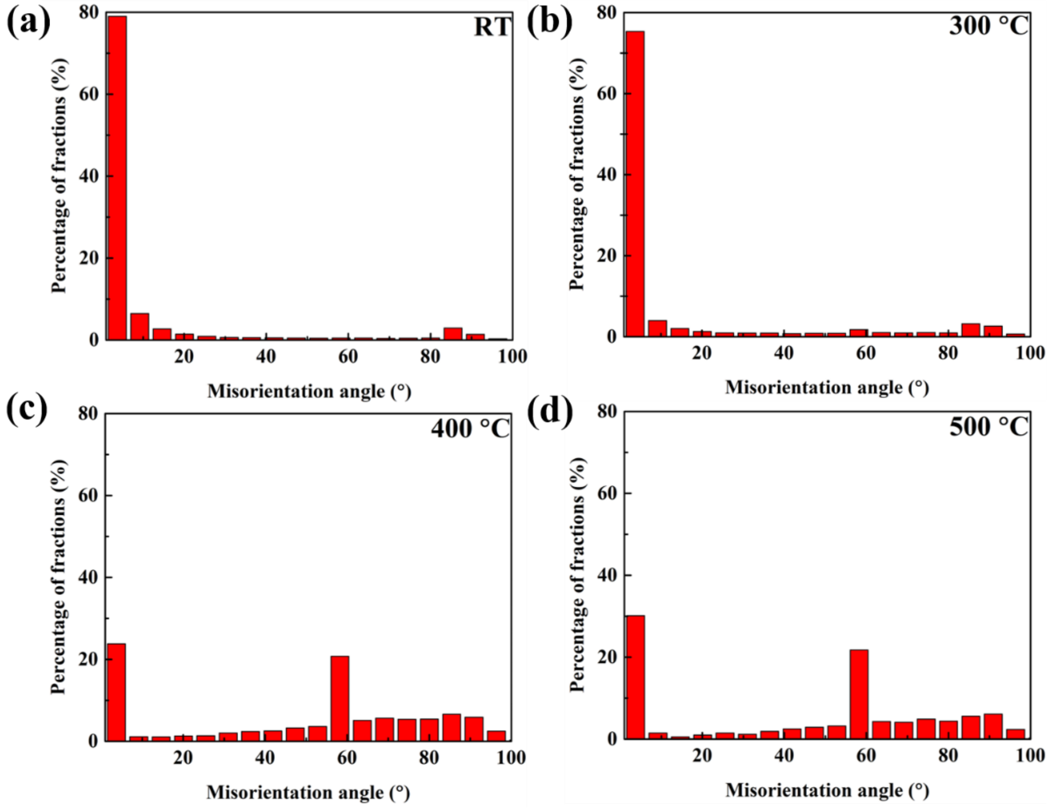

Figure S5. Misorientation angle distribution based on EBSD data at RT, 300, 400, and 500 ℃.
